# Ocular Manifestations of Ebola Virus Disease: An Ophthalmologist's Guide to Prevent Infection and Panic

**DOI:** 10.1155/2015/487073

**Published:** 2015-10-18

**Authors:** Enzo Maria Vingolo, Giuseppe Alessio Messano, Serena Fragiotta, Leopoldo Spadea, Stefano Petti

**Affiliations:** ^1^Ophthalmology Department “Polo Pontino,” Sapienza University of Rome, 04120 Terracina, Italy; ^2^Dental Section, Department of Public Health and Infectious Diseases, Sapienza University of Rome, 00161 Rome, Italy

## Abstract

Ebola virus disease (EVD—formerly known as Ebola hemorrhagic fever) is a severe hemorrhagic fever caused by lipid-enveloped, nonsegmented, negative-stranded RNA viruses belonging to the genus *Ebolavirus*. Case fatality rates may reach up to 76% of infected individuals, making this infection a deadly health problem in the sub-Saharan population. At the moment, there are still no indications on ophthalmological clinical signs and security suggestions for healthcare professionals (doctors and nurses or cooperative persons). This paper provides a short but complete guide to reduce infection risks.

## 1. Introduction

Ebola virus disease (EVD—formerly known as Ebola hemorrhagic fever) is a severe hemorrhagic fever caused by lipid-enveloped, nonsegmented, negative-stranded RNA viruses belonging to the genus* Ebolavirus*. Ebola virus and Marburg virus constitute the family Filoviridae in the order of Mononegavirales. These viruses have characteristic twisted filamentous particles that give the virus family its name. Ebola virus particles have a uniform diameter of 80 nm but can greatly vary in length, 1 *μ*m or even longer [1].

The first reported cases of EVD occurred in 1976 in southern South Sudan (former Sudan) and in northern Democratic Republic of the Congo (former Zaire). The name Ebola corresponds to the name of a small river located in the endemic area (northwestern Zaire) [2, 3]. Five different species of Ebola viruses are recognised,* Bundibugyo*,* Zaire*,* Sudan*,* Reston*, and* Taï Forest*. The three first species caused epidemics in Africa with high case fatality rates, while no human death due to the two other species has ever been reported [4].

EVD is a classic zoonosis with persistence of the virus in reservoir species which live in endemic areas. Viral transmission chain was unveiled after a long absence of epidemic EVD. Animal trapping missions were carried out in areas where several cases had occurred. Three species of fruit bats were found asymptomatically and naturally infected with Ebola virus, thus suggesting that these animals are the natural reservoir [5]. Such idea was corroborated by experimental studies which reported successful Ebola virus infection transmission in fruit bats [6] and by the identification of Marburg virus in fruit bats [7]. Indeed, virus is present in saliva of chronically and asymptomatically infected fruit bats, which expose other species through direct transmission via bites. Indirect transmission also is postulated, as infected bats drop to the ground partially eaten fruit, which may be eaten by nonhuman primates. This process would hypothetically promote their infection. Whatever the route of infection is, it is necessary to amplify the number of infected individuals of the secondary host species to promote the emergence of human disease. A single infected human does not make an epidemic which is, however, promoted by the persistence of prerequisites for Ebola virus infection transmission [5].

Ebola virus infects human monocytes and induces the production of virus-cell particles that result in the loss of endothelial barrier function. In addition, infected monocytes release proinflammatory cytokines and chemokines, which further decrease endothelial barrier function [8]. Animal and in vitro studies show that the Ebola virus has a number of physical and biological mechanisms to evade host innate and acquired humoral and cellular immune responses. These mechanisms promote rapid virus dissemination and replication [9].

Accurate description of the clinical manifestations of EVD was made, for the first time, during the outbreak that occurred in 1995 in the Democratic Republic of the Congo, because it secondarily affected a large number of healthcare workers. The evolution of the disease was followed from the beginning and differences between survivors and fatal cases were directly observed. Although the incubation period is thought to range between two and twenty-one days, the mean observed incubation period among secondary cases was six days, ranging between five and eight days after the contact. Common EVD manifestations are fever, asthenia, diarrhea, abdominal pain, myalgia, arthralgia, melena, sore throat, conjunctival injection, and rush. Fatal cases also show anuria, shock, tachypnea, hiccups, dysesthesia, and bleeding (e.g., gum bleeding, epistaxis, bleeding on injection sites, hematuria, melena, hematemesis, and hemoptysis). Survivors show severe asthenia, tinnitus, hearing loss, coughing, vision loss, conjunctivitis, and uveitis [10].

## 2. Epidemiology

Case fatality rate, assessed during outbreaks that occurred between 1976 and 2012, is 65% (95% confidence interval, 55–75%). Case fatality rates of the single species are 76% (95% confidence interval, 63–87%), 55% (95% confidence interval, 50–59%), and 37% (95% confidence interval, 14–63%) for the* Zaire*,* Sudan*, and* Bundibugyo* species, respectively [11].

The current outbreak is the largest known. It started in February 2014 in Guinea and spread into Liberia in March and Sierra Leone in May, followed by other countries. Its exponential expansion in the first period raised great public health concern. The peculiar characteristic of the Ebola virus strain responsible for the last outbreak is that, given its genetic closeness with the two strains responsible for the most recent outbreaks, it probably derived from both these specimens. However, it underwent human adaptive mutations, which ultimately increased the person-to-person transmission [12]. Indeed, on March 4, 2015, the World Health Organization reported 24,000 cases and 10,000 deaths. These figures show without any doubt that this is the most widespread and long-term outbreak since the discovery of the Ebola virus [13].

EVD may be characterized by high fever, arthralgia, and mild coagulopathy or be asymptomatic at all. Indeed, during various outbreaks, prevalence of individuals with high levels of anti-Ebola virus IgG was relatively high, ranging between 1 and 6% in villages and 20 and 30% in the forest (Table 1) [2, 3, 14–18]. Only a fraction of these individuals reported fever in the days of the outbreak, while the majority were asymptomatic. Early during the infectious process, asymptomatic individuals show strong inflammatory response, which causes virus clearance. Conversely, the delayed inflammatory response, observed among symptomatic subjects, is at the basis of the most severe hemorrhagic symptoms of EVD [19].

## 3. Human-to-Human Ebola Virus Infection Transmission

Human-to-human transmission of EVD is reported in large outbreaks which occur in remote locations, where proper medical, public health, transportation, and communication infrastructure are limited. Ebola virus infection and, sometimes, amplification in hospital settings are frequently described. Widespread transmission events typically involve hospital settings where protective equipment is limited or unavailable, thus suggesting that transmission in healthcare settings can be largely prevented by basic infection control precautions and proper disposal of contaminated items. Indeed, the infectious nature of person-to-person transmission is not efficient. It is limited to direct contact, through broken skin or mucous membranes, with blood, secretions, organs, or other bodily fluids of infected people, and with contaminated surfaces and materials (e.g., bedding and clothing). During outbreaks, there can be several secondary cases (i.e., infection transmission from the index case) and few tertiary cases (i.e., infection transmissions from the secondary cases) [19].

According to epidemiologic studies among households, most secondary cases have direct physical contact with blood, organs, or bodily fluids of diseased persons or cadavers [18, 20]. However, since some individuals do not report direct contact with EVD patients other routes of transmission are plausible [21–23]. An exhaustive example of transmission tree was performed with all cases and contacts reported from Nigeria during the 2014 outbreak. The index case was an infected individual coming from Liberia and EVD was diagnosed after three days. Since then, specific preventive measures were applied that resulted in the end of the outbreak within few weeks. A total of 898 contacts were subsequently identified and linked to the index case, split into 351 primary/secondary contacts and 547 tertiary/higher-order contacts. The outbreak resulted in 19 cases; more than half of them were healthcare workers. The index patient generated twelve secondary cases, while five tertiary and two quaternary cases occurred. Therefore, only 2% of all contacts developed Ebola virus infection or EVD [24].

A transmission route that raises public concern is through the air. One study on three healthy rhesus macaques housed in cages located three meters away from cages that housed infected animals found that two of them became infected and developed EVD [25]. Conversely, another similar study performed on two cynomolgus macaques housed in cages placed thirty centimeters away from the cages of infected animals reported that healthy macaques did not develop the infection [26]. These discrepant studies suggest that Ebola virus airborne transmission is unlikely to occur and is probably due to bloody coughing or sneezing with spatter production (i.e., droplets larger than 50–100 *μ*m in size that fall in the environment within 1/100 seconds) that fall within few meters from the source, rather than through nonbloody aerosol and droplet nuclei (i.e., droplets smaller than 50 *μ*m in size that suddenly desiccate and become droplet nuclei, 1–5 *μ*m in size), which may be suspended in air for hours before their sedimentation.

The main problem in assessing human-to-human Ebola virus transmission is that the minimum infectious dose of the microorganisms is unknown. This makes it difficult to assess how much blood or bodily fluids are necessary to develop the infection.

## 4. Ebola Virus Infection Transmission to Healthcare Workers

Healthcare workers are the category at the highest risk of secondary Ebola virus infection. For example, during the 1995 outbreak in the Democratic Republic of the Congo, one-fourth of all cases and the majority of secondary cases were healthcare workers who had provided care to EVD patients without appropriate contact precautions. Only one healthcare worker, who reported inadvertently rubbing her eyes with contaminated gloves, developed EVD despite the implementation of precautions based on Personal Protective Equipment (PPE) [27]. If adequate control measures are properly applied, the risk of infection among healthcare workers is minimal. Indeed, during the 2014 outbreak, most infections developed among healthcare workers occurred before PPE-based measures were applied in hospitals or in emergency departments, where EVD patients might be confused with patients with malaria, typhoid fever, or other tropical diseases characterized by high fever [28]. The situation in Ebola treatment units in countries where the virus is endemic is terrible, with patients who fall out of bed or in delirium and try to crawl out. The environment is extensively contaminated by blood, vomit, and diarrhea; instruments and fomites also are heavily contaminated. The risk for healthcare workers of being infected without PPE is close to 100%; such a risk is, however, minimal with proper PPE [29].

Ebola virus transmission to healthcare workers is due to blood or secretions and organs contaminated by blood. Indeed, Ebola virus serum level in EVD patients is high soon after the onset of symptoms. Such level is as high as 10^8^ viral particles per serum milliliter in fatal cases and 10^6^ in nonfatal cases [30]. Detectable but low virus levels are occasionally reported in tears, saliva, semen, breast milk, and urine of EVD subjects during the acute phase, while the virus is virtually undetectable in convalescent patients. These data suggest that nonbloody bodily fluids are unlikely to transmit the Ebola virus infection [31, 32]. Ebola virus is not detectable in the environment of Ebola treatment units and in medical instruments used on EVD patients where there are no visible blood stains and surface disinfection is routinely performed. Thus, once again, blood is responsible for transmission, while fomites and other environmental surfaces, which are not contaminated by infected blood, are not [31].

These data suggest that Ebola virus transmission through bodily fluids without blood as well as airborne transmission is unlikely. However, since Ebola virus transmission is not completely understood, exceptional precautions, such as pressurized suits with oxygen tanks, are strongly suggested for interventions that generate huge amounts of aerosols (invasive explorations or intubations), for specific situations (e.g., massive hemorrhage), or in laboratories where the Ebola virus is cultivated. Goggles and masks might not even be necessary to speak with conscious patients, as long as a distance of one to two meters or a glass/plastic barrier is placed between the two subjects [33]. The use of extremely sophisticated PPE in African countries where outbreaks occur may be even counterproductive. Indeed, the image of healthcare workers with spectacular protective clothing encourages panic in some communities because they suggest that the only defense against Ebola virus is sophisticated PPE, which is inaccessible to the general population [33]. In addition, extreme PPE raises concerns in the general population regarding malevolent activities, such as intentional killing and stealing blood or body parts, being performed and local healthcare workers may not cooperate in wearing PPE. For this reason, local healthcare workers may not cooperate in wearing any PPE, thus exposing themselves to Ebola virus infection transmission [30].

## 5. What Are the Chances of Someone Infected with Ebola Virus Seeking Ophthalmologic Care? What Are the Effective Infection Control Measures?

It is generally believed that it is unlikely that EVD patients may seek specialized healthcare, as ophthalmologic care is. However, the number of people coming from West Africa to Western countries, such as Europe, USA, and Australia, is not trivial. For example, approximately 850 individuals come from Ebola virus endemic areas to London each month. If it is true that subjects with severe EVD, who are infectious, are likely to seek emergency services and hospitalization rather than other healthcare services [34], asymptomatic individuals and those with mild EVD are largely prevailing (Table 1) and although poorly infectious, they may seek ophthalmologic care, because of their symptoms.

Several international organizations, such as the Centers for Disease Control and Prevention (CDC) (available at http://www.cdc.gov/vhf/ebola/hcp/infection-prevention-and-control-recommendations.html; accessed version updated November 4, 2014) and World Health Organization (WHO) (available at http://www.who.int/csr/disease/ebola/protective-measures-staff/en/; accessed version updated October 3, 2014), have released and periodically update guidelines for infection control directed to healthcare workers who treat EVD confirmed/suspected patients. These measures are based on the available evidence and on the Precautionary Principle [35]. The most important procedures to prevent Ebola virus infection transmission are displayed in Table 2. PPE use and careful management of sharp instruments are highly recommended, since the strength of the evidence of their effectiveness is strong. The control of droplets, nonsharp instruments, and environmental surfaces also is recommended but at a lower degree of strength based on the Precautionary Principle, since airborne transmission and contact transmission without broken skin and mucosae are hypothesized but undemonstrated.

Globally, these measures are practical and are not particularly different from those generally put into practice by the majority of ophthalmologists. They are enough to prevent Ebola virus infection transmission to healthcare workers, staff, and following patients. In addition, they may help prevent panic and anxiety, a prerequisite for safe and good practice.

## 6. Conjunctivitis: A Key Ophthalmologic EVD Symptom

During outbreaks, the risk of dealing with false alarms largely overwhelms the chance that infectious individuals may unexpectedly present at an ophthalmologist office, due to panic generated by the severity of the disease and by misinformation and alarming campaigns through the media [34]. Thus, the key question is whether ophthalmologists are prepared to recognize Ebola virus infected patients.

In endemic areas, primary EVD cases at the initial stages of the disease are undistinguishable from patients affected by malaria (high fever), shigellosis and typhoid fever (diarrhea), and other protozoal, bacterial, and viral zoonoses. However, as long as hemorrhagic fever becomes manifest, the differential diagnosis between Ebola (or Marburg) virus and Lassa fever is to be made and the characteristics of conjunctivitis, one of the main hemorrhagic symptoms, become crucial. Indeed, in Lassa fever conjunctivitis is severe with periorbital swelling and pain, while in EVD conjunctivae are often injected but asymptomatic [2, 10, 36].

The situation is largely different in Western countries. The risk of false alarms is much higher and, therefore, the first issue to consider when evaluating a person for exposure to Ebola virus is to investigate epidemiologic risk factors through the anamnesis. The checklist provided by CDC, available at http://www.cdc.gov/vhf/ebola/exposure/risk-factors-when-evaluating-person-for-exposure.html, is exhaustive enough. The risk is classified into high, some, low, and no. For example, there is no epidemiologic risk if a patient has been in a country where Ebola virus is endemic more than twenty-one days before presenting at the healthcare worker, because twenty-one days is the longer possible incubation period.

On physical examination, conjunctivitis is a key EVD sign. It is typically bilateral, asymptomatic, and nonicteric. In secondary EVD, conjunctivitis is the earlier sign together with influenza-like symptoms (e.g., asthenia and fever) and may appear even 6-7 days before patients seek EVD-related care [37]. Thus, it is possible that these subjects may seek ophthalmologic care before seeking EVD-related care. Persistent and nonhemorrhagic conjunctivitis in EVD patients is a good prognostic factor, while hemorrhagic conjunctivitis is predictive of death within a few days [3, 10, 19, 36–39].

Summarizing, bilateral, asymptomatic, and nonicteric conjunctivitis is one of the earliest and most frequent signs of EVD and has an important prognostic value.

## 7. Uveitis: A Late Ophthalmologic Symptom

A relevant proportion of convalescent patients, as many as 20% according to one survey [40], who may be asymptomatic for up to 1-2 months, may develop uveitis characterized by ocular pain, photophobia, hyperlacrimation, and loss of visual acuity. Uveitis also is reported in patients who recover from Marburg disease after being asymptomatic for at least two months [41]. Pathogenesis of uveitis may be a delayed hypersensitivity reaction to RNA viral antigens. Uveitis can be treated with topical steroids [40].

It is possible that convalescent patients with late-onset uveitis may seek ophthalmologic care. These patients are considered safe and not infectious, although Ebola virus is detectable in tears of acute-phase EVD patients [31]. Nevertheless, PPE, as in Table 2, must always be adopted in patients with ascertained epidemiological risk.

## 8. Conclusion

EVD is typically a zoonosis and outbreaks with human-to-human transmission periodically occur. The severity of this disease with its high fatality rate and its awful hemorrhagic symptoms has been largely emphasized by mass media, thus generating panic in the general population and, most of all, healthcare providers who are the category at highest risk of secondary cases. Yet, false alarms are largely prevailing on true EVD patients. In addition, none of the healthcare workers from Western countries secondarily infected by the Ebola virus developed severe EVD and no tertiary cases occurred (see the CDC website at http://www.cdc.gov/vhf/ebola/outbreaks/2014-west-africa/united-states-imported-case.html). The risk for EVD among ophthalmologists from Western countries is, therefore, minimal. However, it is not impossible that mild, asymptomatic, and convalescent EVD patients may seek ophthalmologic care. Proper anamnesis and physical examination are enough to distinguish between false alarms and potential Ebola virus carriers and, in the latter case, preventive measures are effective in minimizing the risk of transmission.

## Figures and Tables

**Table 1:** Prevalence of individuals with high serum levels of IgG anti-Ebola virus (i.e., immune against EVD), who were not close contacts of EVD patients, during Ebola virus outbreaks.

First author, year	Country	Setting	Prevalence
WHO, 1978a [2]	Sudan	Overall	6%

WHO, 1978b [3]	Zaire	Overall	1%

Baron, 1983 [18]	Sudan	Village	18%

Busico, 1999 [14]	Democratic Republic of the Congo	Village	2.2%

Gonzalez, 2000 [15]	Central African Republic	Village	3.1–3.7%
		Forest	1.9–12.1%

Becquart, 2010 [16]	Gabon	Village	2–7–12.4%
		Forest	18.4–21.2%

Nkoghe, 2011 [17]	Gabon	Overall	15.3%
		Deep forest	5.0–32.4%

**Table 2:** Summary of the main recommendations for the treatment of suspected Ebola virus infected patients in ophthalmologic care settings (from CDC and WHO websites).

Personal Protective Equipment (PPE)	Ebola virus infection may be transmitted through broken skin and mucosae	Gown, gloves (possibly double gloves), surgical mask, eye visor/goggles, or face shield to protect conjunctival, nasal, and oral mucosae at the same time Choose PPE of exact size Gloves or other PPE that becomes contaminated by blood or bodily fluids must be cleaned or changed before touching other instruments or surfaces Gloved/ungloved hand hygiene. Use alcohol-based hand rub or soap and running water	Strength of the evidence High

Sharp instruments	Sharp instruments are extremely dangerous because they become contaminated by blood or bodily fluids and may break skin/mucosae even if protected by PPE	Use of needles and other sharp instruments must be limited. These instruments must be handled with extreme care and disposed after use in dedicated seal containers	Strength of the evidence High

Droplets	Airborne transmission is not demonstrated Preventive measures are recommended under the Precautionary Principle	If aerosol generating procedures or events, such as coughing or sputum induction, occur, the use of powered air-purifying respirator or respirator (FFP2 or EN certified equivalent or US NIOSH-certified N95) is recommended	Strength of the evidence Low

Nonsharp instruments	Indirect transmission through nonsharp contaminated instruments is not demonstrated Preventive measures are recommended under the Precautionary Principle	Use of disposable medical equipment is recommended or, alternatively, nondisposable medical equipment must be cleaned and disinfected after use according to manufacturer's instructions	Strength of the evidence Low

Environmental surfaces	Environmental surfaces do not pose a risk of infection. However, Ebola virus is nonenveloped and is able to survive in the environment for long time Preventive measures regarding surfaces visibly contaminated with blood and bodily fluids are recommended under the Precautionary Principle	Use of standard hospital detergents and disinfectants (e.g., 0.5% chlorine solution or a solution containing 5000 ppm available free chlorine), preceded by cleaning to prevent inactivation of disinfectants by organic matter, is recommended	Strength of the evidence Low
